# NF-κB Signaling and IL-4 Signaling Regulate SATB1 Expression via Alternative Promoter Usage During Th2 Differentiation

**DOI:** 10.3389/fimmu.2019.00667

**Published:** 2019-04-02

**Authors:** Satyajeet P. Khare, Ankitha Shetty, Rahul Biradar, Indumathi Patta, Zhi Jane Chen, Ameya V. Sathe, Puli Chandramouli Reddy, Riitta Lahesmaa, Sanjeev Galande

**Affiliations:** ^1^Center of Excellence in Epigenetics, Indian Institute of Science Education and Research, Pune, India; ^2^Symbiosis School of Biological Sciences, Pune, India; ^3^Turku Center for Biotechnology, University of Turku and Abo Akademi University, Turku, Finland

**Keywords:** SATB1, alternative promoter, TCR signaling, cytokine signaling, STAT6

## Abstract

SATB1 is a genome organizer protein that is expressed in a lineage specific manner in CD4^+^ T-cells. SATB1 plays a crucial role in expression of multiple genes throughout the thymic development and peripheral differentiation of T cells. Although SATB1 function has been subjected to intense investigation, regulation of *SATB1* gene expression remains poorly understood. Analysis of RNA-seq data revealed multiple transcription start sites at the upstream regulatory region of *SATB1*. We further demonstrated that *SATB1* gene is expressed via alternative promoters during T-helper (Th) cell differentiation. The proximal promoter “P1” is used more by the naïve and activated CD4^+^ T-cells whereas the middle “P2” and the distal “P3” promoters are used at a significantly higher level by polarized T-helper cells. Cytokine and TCR signaling play crucial roles toward *SATB1* alternative promoter usage. Under Th2 polarization conditions, transcription factor STAT6, which operates downstream of the cytokine signaling binds to the P2 and P3 promoters. Genetic perturbation by knockout and chemical inhibition of STAT6 activation resulted in the loss of P2 and P3 promoter activity. Moreover, chemical inhibition of activation of NF-**κ**B, a transcription factor that operates downstream of the TCR signaling, also resulted in reduced P2 and P3 promoter usage. Furthermore, usage of the P1 promoter correlated with lower SATB1 protein expression whereas P2 and P3 promoter usage correlated with higher SATB1 protein expression. Thus, the promoter switch might play a crucial role in fine-tuning of SATB1 protein expression in a cell type specific manner.

## Introduction

T-cells constitute the cell mediated, adaptive, immune system component in jawed vertebrates. T-cells develop in thymus and differentiate in periphery. The common lymphoid progenitor (CLP) cells develop in thymus into CD4 and CD8 single positive T-cells in a series of steps orchestrated by transcription factor network. The CD4^+^ T-cells which enter peripheral blood further undergo differentiation into one of many functionally distinct T-helper cell subtypes depending on the antigen stimulus and cytokine environment [reviewed in (1, 2)]. Naïve CD4^+^ T-cells respond to pro-inflammatory cytokines IL-12 and IFN-γ and differentiate along Th1 lineage aided by the master regulatory transcription factor T-bet. In contrast, IL-4 directs the differentiation of naïve cells toward the Th2 lineage via the transcription factor GATA-3. Similarly, IL6/TGFβ skew the differentiation of naïve cells toward Th17 lineage via the master regulatory transcription factor RORγT [also reviewed in (3)].

Special AT-rich sequence Binding protein 1 (SATB1) (4), plays a crucial role in the development of T-cells in the thymus as well as their differentiation in the periphery [reviewed in (5)]. In the periphery, SATB1 is expressed by T-helper cells where it activates genes in a locus-specific manner (6, 7). The importance of SATB1 in T-cells is underscored by the observation that *Satb1*-KO animals exhibit arrested thymic development (8). Recently, SATB1 was shown to be essential in specifying T-lymphocyte subsets by directing lineage-specific transcription programs (9).

In Th2 cells, SATB1 regulates expression of GATA3 in a Wnt/β-catenin signaling-dependent manner. Upon Wnt signaling, β-catenin translocates to the nucleus and binds to SATB1 to de-repress a cascade of genes crucial in differentiation (10). SATB1 also regulates downstream production of IL-5 cytokine by direct binding to the *IL-5* promoter (7, 10). In contrast, during regulatory T (Treg) cell differentiation downregulation of SATB1 is essential (11). Treg cells are essential for immune tolerance. Treg cells respond to and secrete the cytokine TGF-β, express the master regulator transcription factor FOXP-3. FOXP-3 represses *SATB1* transcriptionally by regulating its expression and post-transcriptionally by upregulating microRNAs that target 3' UTR of the SATB1 transcripts (11, 12). Interestingly, SATB1 is expressed at the Treg precursor stage of development and plays a crucial role in the lineage specification of Treg cells in the thymus (13).

Despite the importance of SATB1 in T-cell development and function, the mechanism that regulates its expression in T-helper cells remains poorly understood. In thymocytes, *SATB1* gene is dynamically expressed throughout all the stages. The T-cell receptor (TCR) signaling has been shown to play an important role in *SATB1* gene expression during early thymocyte development (14). Specifically, the transcription factor GATA-3 was found to directly regulate SATB1 expression in developing thymocytes by binding to the upstream regulatory region (14). Analysis of publicly available T-cell transcriptome data resulted in identification of a large regulatory region at the *SATB1* gene locus. This large regulatory region codes for multiple *SATB1* mRNA isoforms that differ in the transcription start sites corresponding to promoters. These isoforms that result from alternative promoter (AP) usage, differ in the sequence of the 5' UTR and splicing of the first exon that harbors them. Alternative promoters play crucial role in gene regulation in the determination of cell fate and function. APs allow diversification of transcriptional regulation enabling expression in various cell lineages and developmental stages. Use of APs results in mRNA isoforms that differ in the sequence of 5' UTRs that are crucial for post-transcriptional regulation [reviewed in (15)]. With this background, we studied the role of alternative promoters in *SATB1* expression during T-helper cell differentiation.

Here, we show a complex mechanism of SATB1 regulation during peripheral T-helper differentiation. We found that *SATB1* gene expression is regulated via alternative promoters (proximal P1, middle P2, and distal P3) during peripheral differentiation of CD4^+^ T-cells. The helper T-cells rely on P2 and P3 promoter usage whereas activated T-cells and Treg cells preferentially use the P1 promoter, suggesting the importance of pro-inflammatory cytokines in promoter switching. Experiments performed using a Jurkat cell line based system suggested a crucial role of TCR signaling in P2 and P3 promoter usage. We identified STAT family of transcription factors that operate downstream of cytokine signaling and NF-κB that operates downstream of the TCR signaling as regulators of *SATB1* P2 and P3 promoter usage. Finally, we find differential correlation between *SATB1* isoforms that result from alternative promoter usage and SATB1 protein expression suggesting possible role of alternative promoters in regulation of protein expression.

## Materials and Methods

### RNA-Seq Analysis

Publicly available human CD4^+^ T-cell polyA RNA-Seq datasets [E-MTAB-2319 (16), GSE35871 (17), and GSE71645 (18)] were analyzed to identify SATB1 transcripts in various CD4^+^ primary T-cells and cell-lines. In brief, reads were aligned to reference human genome assembly [hg38, Gencode (19)] using HiSAT2. Transcripts were assembled and merged using Stringtie (20). Merged transcriptome assembly was visualized on IGV Genome Browser (21). CpG island track was downloaded from UCSC genome browser for the hg38 genome assembly and was also uploaded onto the genome browser (22). *SATB1* expression was analyzed in Th2 cells and induced Treg (iTreg) cells using featureCounts (23) and DESeq2 (24). Exon expression was analyzed by generating an exon-count matrix. The GlmQLFit test in EdgeR was applied for differential expression analysis (25). Normalized exon-counts were converted to FPKM for expression plot. Statistical significance of the number of overlapping differentially expressed genes between Jurkat cells and primary T-cells was tested using two-tailed hypergeometric test (26). Junction reads between *SATB1* alternative first exons and second exon were plotted using bam files on the IGV genome browser.

### *In vitro* Differentiation of Naïve CD4^+^ T-Cells and Inhibitor Treatment

Human naïve CD4^+^ T-cells were isolated from total peripheral blood mononuclear cells (PBMCs) by magnetic bead based negative selection (130-094-131, Miltenyi Biotec). In brief, ~100 mL human peripheral blood samples were collected from healthy volunteers. Blood samples were diluted in PBS and subjected to Ficoll Paque (17-1440-03, GE Healthcare) density gradient centrifugation for separation of PBMCs. Purity of naïve CD4^+^ T-cells was confirmed and their *in vitro* differentiation was confirmed by flow cytometry analysis (16). In brief, naïve CD4^+^ T-cells were plated at 1 million/ml density in complete RPMI medium. For non-specific activation, naïve cells were incubated in the presence of 2 μg/ml anti-CD3 (130-093-387, Miltenyi Biotec), 2 μg/ml anti-CD28 (130-093-375, Miltenyi Biotec). For Th2 differentiation, naive cells were incubated in presence of 2 μg/ml anti-CD3, 2 μg/ml anti-CD28, 10 ng/ml IL-4 (130-093-915, Miltenyi Biotec), 10 μg/ml anti-IFNγ (130-095-743, Miltenyi Biotec), and 10 μg/ml anti-IL-12 (130-095-755, Miltenyi Biotec). Cells were harvested after 72–96 h of incubation. Th2 differentiation was confirmed by staining for intracellular IL-4 using anti-IL4 PE antibody (12-7049-42, eBiosciences). Naïve CD4^+^, Th0, and Th2 cells were subjected to RNA and protein isolation followed by qRT-PCR and Western blot analysis.

For studying the effect of NF-κB transcription factor on *SATB1* alternative promoter expression, naïve CD4^+^ cells were subjected to non-specific activation or differentiation for 48–72 h followed by treatment with 6 μM NF-κB inhibitor (CID2858522, Tocris) for another 24 h. The cells were then harvested and subjected to either qRT-PCR analysis for *SATB1* alternative promoter usage or Western blot.

For studies in mice, spleens were dissected from wild type (WT) and *Stat4* or *Stat6* knockout (KO) animals (Jackson Laboratories, Bar Harbor, ME, USA). Cells were extracted from tissues and were cleared using cell strainer (352340, Corning). Cells were then subjected to naïve CD4^+^ T-cell isolation by negative selection using magnetic beads (CD4^+^CD62L^+^ T cell Isolation Kit II, Miltenyi Biotec, 130-093-227). Similar to human samples, a fraction of naïve CD4^+^ T-cells was activated in presence of plate bound anti-Cd28 500 ng/ml (BD 553295), anti-CD3 1 μg/ml (BD 553238), IL-2 (R&D Systems 419 402-ML), or subjected to *in vitro* Th2 differentiation for 4 days in additional presence of Il-4 10 ng/ml (R&D Systems 404-ML), anti-Ifnγ 10 μg/ml (BD 557530), and anti-Il-12 10 μg/ml (BD 554475). A fraction of cells from WT and knockout animals were subjected to Gata3 (BD 560074), Ifn-γ (BD 554411), and Satb1 (BD 562378) staining to confirm polarization. The other fraction of cells was used for RNA isolation followed by qRT-PCR analysis.

### RNA Sequencing and qRT-PCR Analysis

Total RNA was extracted using RNeasy Mini Kit (Qiagen, #74106). Isolated RNA were either subjected to PolyA enrichment using MicroPoly(A) Puris Kit (Thermo #AM1919) followed by sequencing (Accession number PRJNA503398) or cDNA synthesis using random hexamer primers for differential gene expression analysis. Alternative first exon specific forward primers (Exon 1a, 1b, and 1c corresponding to putative promoters P1, P2, and P3) were designed and used with Exon 2 specific reverse primer. Gene expression (*SATB1, GATA3, IL2RA*, 18s rRNA) analysis was also carried out using specific primers. Primer sequences used in qRT-PCR analysis are summarized in Supplementary Table 1, Supplementary Information.

### ChIP-Seq Analysis

Publicly available mouse dataset for Stat6 ChIP-seq in Th2 and Stat4 ChIP-seq in Th1 [GSE22105 (27)], and H3K4me3 ChIP-Seq in naïve, Th2 and Th1 cells [GSE14254 (28)] were analyzed for binding on the mouse *Satb1* locus. In brief, ChIP-seq reads were aligned to mouse Gencode mm10 genome assembly (19) using Bowtie2 (version 4.8.4) (29) and BWA (version 0.7.12) (30) for Stat4 or Stat6 ChIP-Seq and H3K4me3 ChIP-Seq datasets, respectively. For Stat4 and Stat6 ChIP-Seq, aligned files were subjected to peak calling using MACS (31). The bed files and the bigwig files were visualized on the IGV genome browser.

### Jurkat Cell Culture and Treatments

Jurkat E6.1 cells were obtained from ATCC (TIB-152). Cells were cultured in RPMI 1640 complete medium (10% FBS) as per the ATCC guidelines. Cells were seeded at 0.2 million/ml density 1 day prior to the activation. Jurkat cells were activated with 0.1 μg/ml Phorbol 12-myristate 13-acetate (PMA) (P1585, Sigma), 1 μM Ionomycin (I0634, Sigma), and/or 50 ng/ml IL-4 (130095373, Miltenyi Biotec) as indicated. The cells were harvested 48 h after treatment. To dissect out the role of transcription factors in regulation of *SATB1* promoters, Jurkat cells were treated with specific inhibitors 24 h before they were harvested. Following specific inhibitors were used−5 and 20 μM JAK3 inhibitor (420122, Calbiochem), and 6 μM NF-κB inhibitor (CID2858522, Tocris). After harvesting, Jurkat cells were subjected either to qRT-PCR analysis for *SATB1* alternative promoter usage or for expression of various other genes (*IL2RA, GATA3*) or were subjected to western blot analysis.

### Western Blot Analysis

Cell lysates were prepared in RIPA buffer (150 mM NaCl, 1% IGEPAL, 0.5% Na-deoxycholate, 0.1% SDS, 50 mM Tris pH8) with protease and phosphatase inhibitors. Protein amounts were estimated by BCA method (Thermo Scientific # 23227). Equal amounts of protein were electrophoresed on a 10% SDS-polyacrylamide gel and transferred onto a PVDF membrane. Western blot analysis was performed using the following primary antibodies: rabbit anti-pSTAT6 (#9361, Cell Signaling Technology), rabbit total anti-STAT6 (#9362, Cell Signaling Technology), rabbit anti-SATB1 (#3650, Cell Signaling Technology), anti-GATA-3 (Abcam, ab106625), mouse anti-β-Actin (ac004, Abclonal), mouse anti-γ-Tubulin (#T6557, Sigma), and appropriate anti-mouse and anti-rabbit secondary antibodies. Densitometry analysis was performed using ImageJ (v 1.5.1) (32).

### Statistical Analysis

Gene expression values were normalized to the Control group as indicated. Student's *t*-test was applied for two-group comparisons. For multiple group comparisons, one-way ANOVA was performed with *post-hoc* Bonferroni correction using commercial software (Prism 5.0a, GraphPad).

## Results

### SATB1 Uses Alternative Promoters During Th2 Differentiation

**Figure 1:**
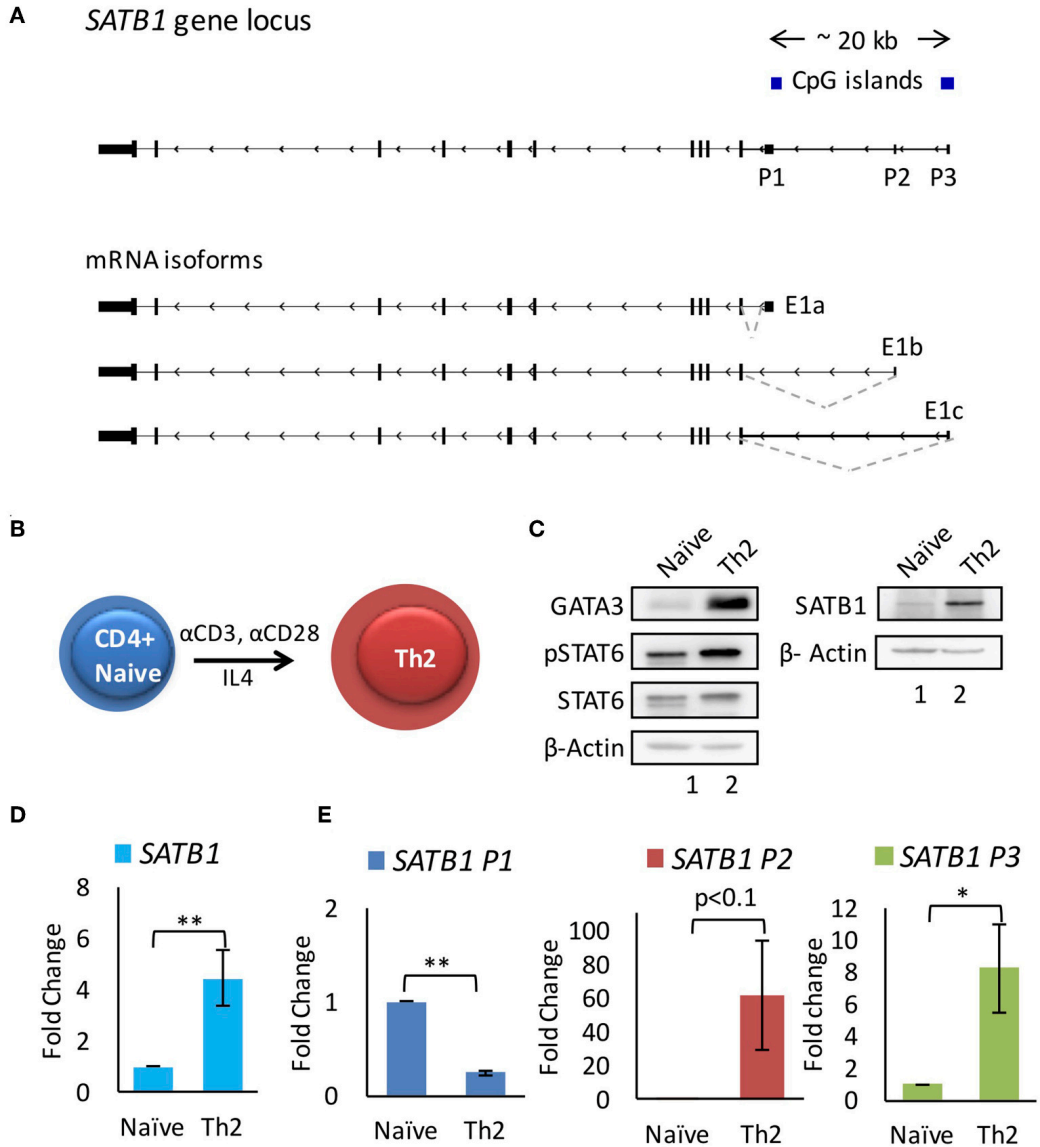
*SATB1* alternative promoter usage during T-helper 2 (Th2) cell differentiation. **(A)** *SATB1* isoforms in CD4^+^ T-cells identified by RNA-Seq analysis. Three mRNA isoforms of *SATB1* with alternative first exons (E1a, E1b, and E1c) that correspond to the usage of three alternative promoters (P1, P2, and P3, respectively). **(B)** Schematic of *in vitro* differentiation of naïve CD4^+^ T-cells into Th2 cells. Naive CD4+ cells were treated with anti-CD3 and anti-CD28 along with IL4 for 96 h to induce Th2 differentiation. **(C)** Immunoblot assay performed as mentioned in methods using the antibody against GATA3, phospho-STAT6 (pSTAT6), total STAT6 and SATB1 for naïve CD4^+^ and Th2 cells. Increase in expression of GATA3, pSTAT6, SATB1 confirms the differentiation of naive CD4^+^ cells into Th2 cells. **(D,E)** qRT-PCR analysis for total *SATB1* expression and *SATB1* alternative promoter usage in naive and differentiated Th2 cells. A significant increase in expression of SATB1 is observed which corresponds to increased usage of *SATB1* P2 and P3 promoter. Error bars indicate SEM. (*N* = 6; ^*^ < 0.05, ^**^ < 0.01); *P*-values were calculated using Student's *t*-test.

We performed analysis on publicly available RNA-Seq datasets for primary CD4^+^ T-cells and cell lines [E-MTAB-2319 (16), GSE35871 (17) and GSE71645 (18)] and found presence of three mRNA isoforms expressed at the *SATB1* locus. These isoforms differ in their transcription start site (TSS) and therefore 5' untranslated region (5'UTR). Interestingly, these isoforms do not differ in the coding DNA sequence (CDS) and thus seem to code for identical SATB1 protein sequence. The three transcription start sites encompass a ~20 Kb regulatory region. We marked the regions around the TSSs as putative promoters (P1 proximal, P2 middle, and P3 distal) (Figure 1A). We checked the expression of these three isoforms in human naïve CD4^+^ T-cells and those subjected to *in vitro* differentiation into Th2 phenotype (Figure 1B) by quantitative real-time PCR (qRT-PCR) analysis. Naïve cell isolation and Th2 differentiation was confirmed by flow cytometry analysis for CD4 and IL-4 expression, western blot analysis for expression of Gata3 and activated Stat6 (pStat6) (Supplementary Figures 1A–D, Figure 1C). Expectedly, total SATB1 protein and gene expression also increased during Th2 differentiation (Figures 1C,D). The proximal promoter P1 transcripts showed decrease in expression whereas the P2 and P3 promoter transcripts showed significant increase in expression upon Th2 differentiation (Figure 1E).

### Stat6 and Stat4 Regulate *Satb1* P2 Promoter Usage

**Figure 2:**
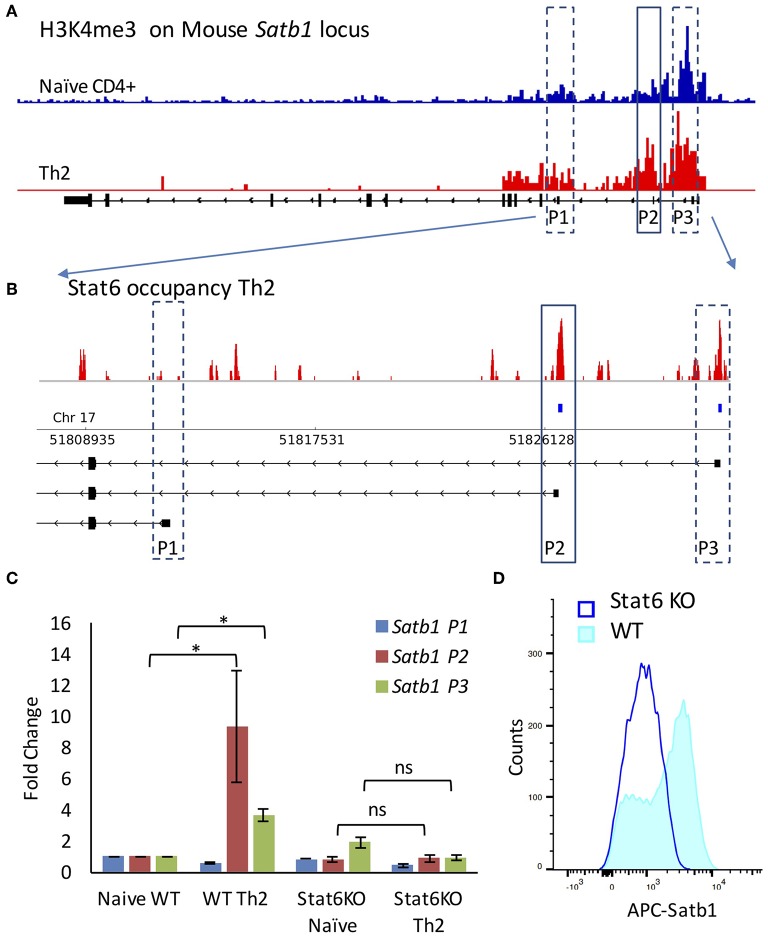
Stat6 regulates P2 promoter usage *in vivo*. **(A)** ChIP-Seq analysis of H3K4me3 levels on *Satb1* locus in naive CD4+ and Th2 cells (mouse mm10 genome assembly) performed as mentioned in “Materials and methods”. H3K4me3 marks are enriched at the P2 and P3 promoter regions in Th2 cells as compared to naive CD4^+^ T-cells. **(B)** ChIP-Seq analysis of Stat6 occupancy at the *Satb1* alternative promoters in Th2 cells (enlarged view of *Satb1* regulatory region). Stat6 ChIP-Seq aligned reads (first track) and significant peaks (second track) along with *Satb1* alternative promoters in mouse (mm10 genome assembly). Stat6 binds to the *Satb1* P2 promoter in T-helper cells. **(C)** qRT-PCR analysis of *Satb1* alternative promoter usage (P1, P2, and P3) in naive CD4^+^ and Th2 cells performed in WT and *Stat6* KO mice, respectively. Error bars represent SEM (*N* = 4); *P*-values were calculated using one-way ANOVA (^*^ < 0.05). *Stat6* KO adversely affects *Satb1* alternative promoter usage. Unlike the wild type animals, no significant increase is observed in *Satb1* P2 and P3 promoter usage in cells from *Stat6* KO animals subjected to Th2 differentiation conditions. **(D)** Flow cytometry analysis for Satb1 protein expression in wild-type and *Stat6* KO, respectively under Th2 differentiating conditions. Satb1 protein expression is not enhanced when naive T-cells from *Stat6* KO animals are subjected to Th2 differentiation conditions.

We performed Chromatin Immunoprecipitation sequencing (ChIP-Seq) analysis on public datasets [GSE22105 (27)] to study the histone modifications typically associated with transcription activation [H3K4me3 (33)] upon Th2 differentiation. We found that as compared to naïve CD4^+^ T-cells, the Th2 cells showed increase in H3K4me3 marks on P2 and P3 promoter (Figure 2A). We then analyzed the ChIP-Seq data for the master regulator transcription factors of Th2 differentiation, such as Gata3 (34) [GSE20898 (35)] and Stat6 (36) [GSE14254 (28)] for their involvement in *Satb1* promoter regulation. We found that Stat6 exhibited differential occupancy on mouse *Satb1* promoters. Interestingly, Stat6 occupied P2 and P3 promoter regions but not the P1 promoter (Figure 2B). To study the importance of Stat6 binding in the regulation of alternative promoter usage, we used *Stat6* knockout (KO) mice. Naïve CD4^+^ T-cells isolated from spleens of wild-type and *Stat6*-KO mice were subjected to Th2 differentiation conditions. *Satb1* isoform expression analysis by qRT-PCR suggested that P2 promoter usage was significantly affected in *Stat6*-KO mice (Figure 2C). *Stat6*-KO also resulted in significant decrease in *Satb1* protein levels (Figure 2D) suggesting a significant contribution of P2 and P3 promoters toward protein expression.

Increase in Satb1 expression has also been observed in other T-helper subtypes (Supplementary Figure 3). Activation of cytokine signaling and Stat family of transcription factors is a property shared by all T-helper cells [reviewed in (37)]. For example, Stat4 transcription factor plays a crucial role in differentiation of naïve CD4^+^ T-cells into Th1 phenotype (38). We analyzed H3K4me3 marks and Stat4 occupancy at the *Satb1* locus in Th1 cells. Similar to Th2, Th1 cells also showed an enrichment of both H3K4me3 marks and Stat4 occupancy at the P2 promoter. When naïve cells isolated from *Stat4*-KO mice were subjected to Th1 differentiation conditions, they failed to show an increase in P2 promoter usage, unlike the wild-type animals (Supplementary Figure 4).

**Figure 3:**
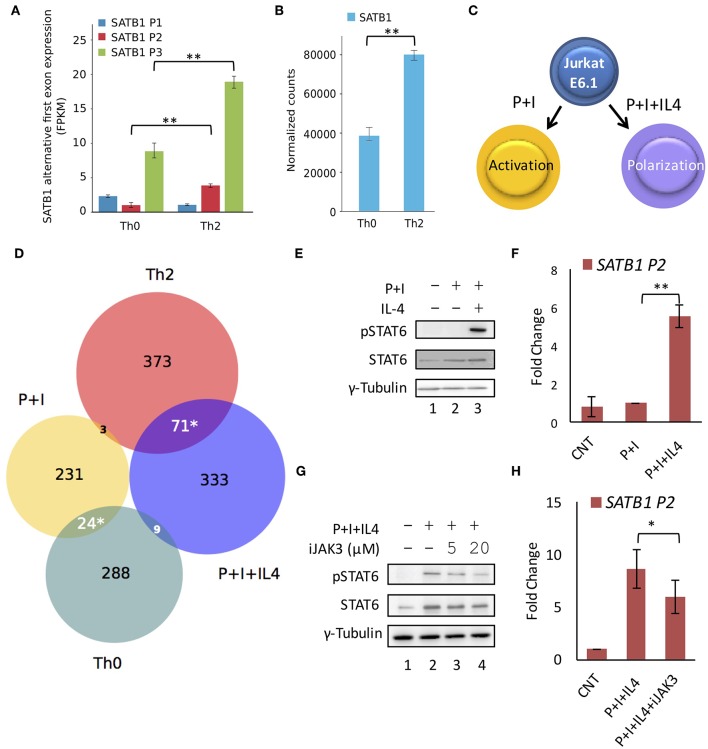
STAT6 regulates P2 promoter usage *in vitro*. RNA-Seq analysis of available data (GSE71645) for **(A)** expression of *SATB1* alternative first exons and **(B)** total *SATB1* expression. Expression analysis confirms higher usage of *SATB1* P2 and P3 promoters and higher total *SATB1* expression in human Th2 cells as compared to Th0 cells. Error bars represent min-max values of FPKM and normalized counts, respectively (*N* = 3); *P*-values calculated using EdgeR and DESeq2 respectively. **(C)** Schematic for the treatment of Jurkat cells under activating (PMA+Ionomycin; P+I) and polarizing conditions (+IL4). **(D)** Venn diagram showing an overlap of differentially expressed genes obtained by RNA-seq analysis between Th0 vs. Th2 cells and P+I vs. P+I+IL4 treated Jurkat cells. Significant overlap is observed between genes expressed higher in Th2 and P+I+IL4 treated Jurkat cells suggesting that Jurkat cell line-based model mimics Th2 differentiation condition. Significance of overlap was calculated using two-tailed hypergeometric test. **(E)** Immunoblot analysis confirms STAT6 activation (pSTAT6) only in Jurkat cells subjected to polarizing conditions. γ-Tubulin was used as loading control. **(F)** qRT-PCR analysis for *SATB1* P2 promoter usage in Jurkat cells activated with TCR and cytokine signaling. A significant increase in expression is observed in *SATB1* P2 promoter usage in Jurkat cells under polarization conditions (*N* = 5). **(G)** Immunoblot analysis for pSTAT6 levels and **(H)** qRT-PCR analysis for *SATB1* P2 promoter usage in Jurkat cells activated with TCR and cytokine signaling in presence of JAK3 inhibitor. A decrease in pSTAT6 levels and *SATB1* P2 promoter usage is observed upon JAK3 inhibition. Error bar represents SEM (*N* = 3); *P*-values calculated using Student's *t*-test (^*^ < 0.05, ^**^ < 0.01).

Next, we analyzed publicly available transcriptome data [GSE71645 (18)] for the expression of *SATB1* alternative first exons in CD4^+^ T-cells subjected to activation by TCR signaling (Th0) and Th2 polarization conditions. We observed that P2 and P3 promoter usage was higher in Th2 cells as compared to Th0 cells underlining the importance of cytokine signaling in P2 and P3 promoter expression (Figure 3A). Total SATB1 expression was also higher in Th2 cells (Figure 3B).

To further validate the importance of Stat6 in P2 promoter usage, we established an *in vitro* system that mimics Th2 differentiation. We cultured Jurkat cells under conditions that mimic polarization signals (Figure 3C) with an aim to establish a system that permits easier manipulation at the genetic level, which will be required for our future studies. Upon treatment of Jurkat cells with PMA and Ionomycin [that mimic activation of TCR signaling (39)] in the presence of IL-4 cytokine, phospho-Stat6 (pStat6) levels were elevated along with increase in *SATB1* P2 promoter usage (Figures 3E,F). We performed RNA-seq analysis of Jurkat cells treated with activating (P+I) and polarizing (P+I+IL4) conditions and found significant overlap of upregulated genes between Jurkat cells activated in presence of IL-4 and Th2 cells (Figure 3D). Treatment of Jurkat cells subjected to polarizing conditions in the presence of JAK3 inhibitor resulted in decrease in P2 promoter usage in a dose-dependent manner (Figures 3G,H), suggesting causal role of JAK/STAT signaling in *SATB1* P2 and P3 promoter usage. Thus, the results obtained using the chemical inhibitors in the *in vitro* system corroborate those obtained from the *in vivo* experiments with genetic perturbation (knockout animals) and consolidate the role of JAK/STAT signaling in the P2 promoter usage.

### NF-κB Signaling Regulates *SATB1* P2 Promoter Usage

We found that STAT-family of transcription factors downstream of the cytokine signaling positively regulate the *SATB1* P2 promoter expression. However, when Jurkat cells were treated with IL-4 cytokine (in absence of PMA and Ionomycin), no increase in P2 and P3 promoter expression was observed (data not shown). The switch in promoter usage was observed only upon simultaneous activation of cytokine and TCR signaling. This observation does not rule out the role of TCR signaling in *SATB1* alternative promoter usage. TCR signaling is mediated by NFAT transcription factors downstream of the CD3 signaling (40) and the AP-1 (41) and NF-κB (42) transcription factors downstream of the CD28 signaling. Synergistic activation of gene expression by cooperativity of STAT6 and NF-κB transcription factors has been reported (43–45). Therefore we checked if NF-κB and STAT6 co-regulate *SATB1* P2 promoter expression by using a chemical inhibitor of NF-κB activation.

**Figure 4:**
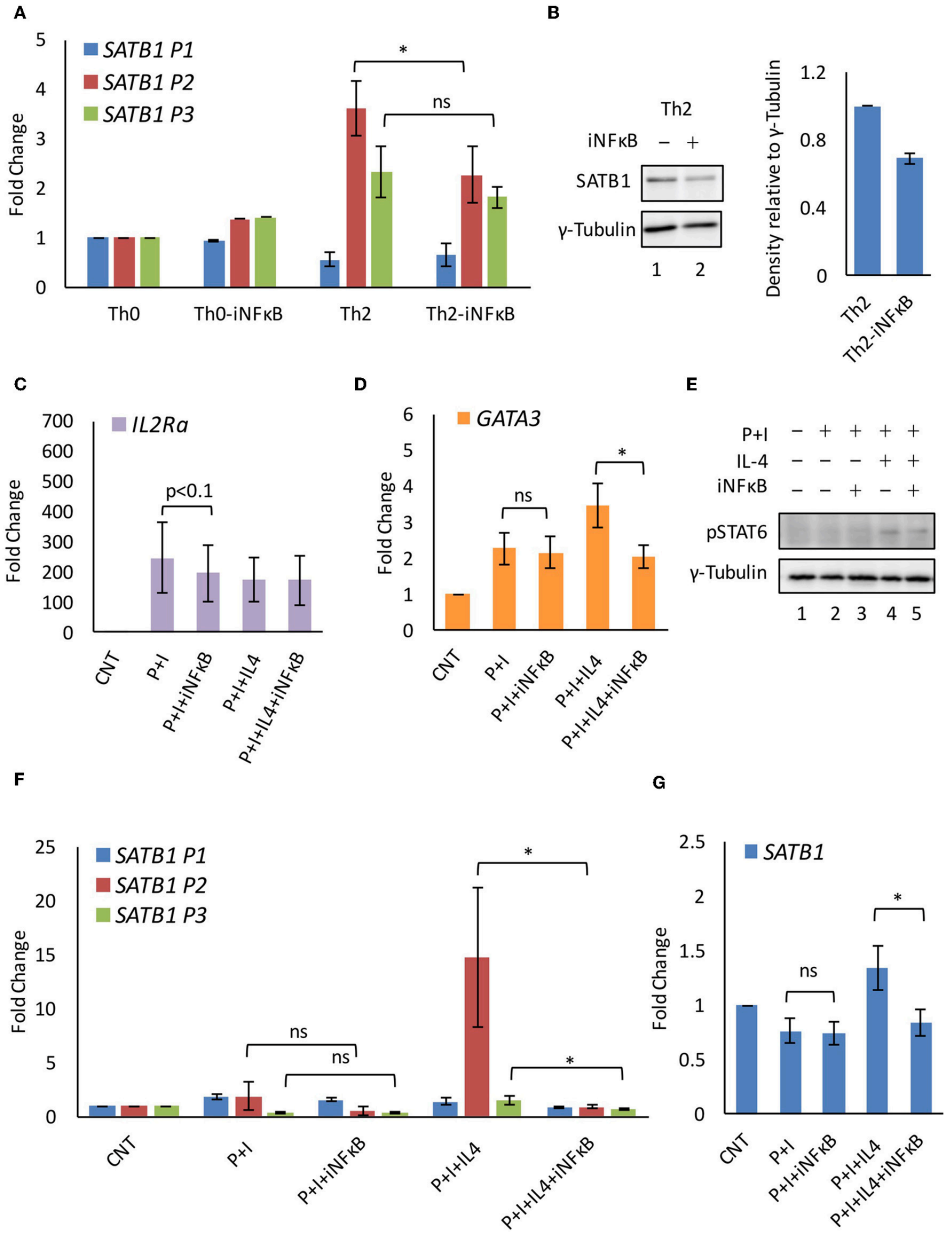
NF-κB regulates *SATB1* P2 promoter expression. **(A)** Quantitative RT-PCR analysis of *SATB1* alternative promoter usage in iNF-κB treated Th0 and Th2 cells. **(B)** Western blot for SATB1 expression in control and iNF-κB treated Th2 cells and densitometry analysis of expression (*N* = 4). Quantitative RT-PCR analysis of NF-κB target genes **(C)** *IL2Ra* and **(D)** *GATA3* confirms NF-κB inhibition in Jurkat cells subjected to activating and polarizing conditions. **(E)** Immunoblot analysis of activated STAT6 (pSTAT6) expression. Quantitative RT-PCR analysis of **(F)** *SATB1* alternative promoter usage and **(G)** total *SATB1* expression upon NF-κB inhibition in Jurkat cells. A significant decrease in *SATB1* P2 and P3 promoter usage was observed upon inhibition of NF-κB in polarized but not in activated Jurkat cells (*N* = 5). Error bar represents SEM and *P*-value calculated using one-way ANOVA (ns, not significant) (^*^ < 0.05).

We subjected naïve human CD4^+^ T-cells isolated from peripheral blood to Th2 differentiation. The differentiating Th2 cells that were treated with inhibitor that specifically affects NF-κB activation downstream of PKCθ resulted in decrease in the P2 promoter usage. This decrease also coincided with decrease in SATB1 protein expression. No similar decrease was observed in the P2 promoter usage in Th0 cells (Figures 4A,B). We tested this observation in Jurkat cells subjected to activating (P+I) and polarizing conditions (P+I+IL4). IL2RA and GATA3 were used as positive controls for NF-κB inhibition in activated (46) and polarized Jurkat cells (47), respectively (Figures 4C,D). We also monitored if NF-κB inhibition affects phosphorylation of STAT6 in polarized Jurkat cells and found no significant difference in pSTAT6 levels upon NF-κB inhibition (Figure 4E). Similar to primary cells, *SATB1* P2 promoter usage was significantly affected when polarized Jurkat cells were treated with the NF-κB inhibitor. Unlike primary cells, usage of the P3 promoter was also affected though not to the same extent as the P2 promoter. Similar to Th0 cells, this inhibition was specific to polarized Jurkat cells since those activated with PMA and Ionomycin did not show any effect on usage of any of the *SATB1* promoters. These results suggested a synergistic activation of *SATB1* P2 promoter usage by IL4 and NF-κB signaling (Figures 4F,G).

### *SATB1* P2 Promoter Usage Is Specific to T-Helper Cells

**Figure 5:**
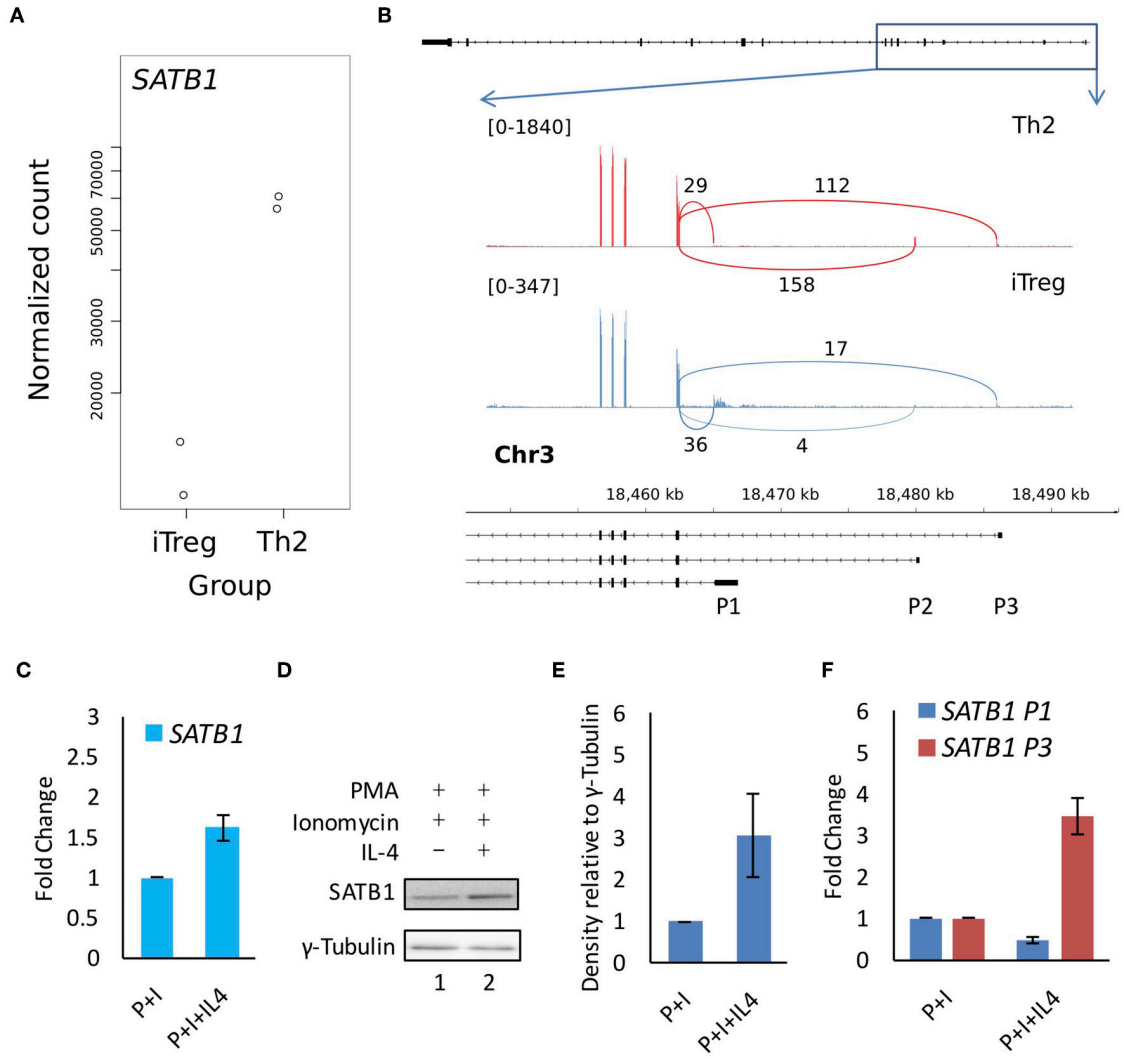
*SATB1* alternative promoter usage in Treg and Th2 cells and correlation with protein expression. Available RNA-seq data (E-MTAB-2319) was analyzed for **(A)** total *SATB1* expression and **(B)** for junction reads at the *SATB1* locus in Th2 and iTreg cells. Total *SATB1* expression is lower but P1 promoter usage is higher in iTreg cells as compared to Th2 cells. **(C)** Quantitative RT-PCR, **(D)** immunoblot, and **(E)** densitometry analysis for SATB1 expression in Jurkat cells subjected to activating and polarizing conditions. **(F)** Quantitative RT-PCR analysis for *SATB1* promoter suggests that the P1 promoter usage showed weak correlation with SATB1 protein expression. Error bar represents SEM (*N* = 3 for **Figures 3C,E,F**).

CD4^+^ T-cells can differentiate into Th cell subtypes or regulatory T-cells. While SATB1 expression has been observed in T-helper cells, SATB1 expression is suppressed in regulatory T-(Treg) cells by both transcriptional and post-transcriptional mechanisms (11). Unlike T-helper cells, Treg cell differentiation is triggered by the anti-inflammatory cytokine TGF-β (48) which leads to the activation of SMAD transcription factors and those downstream of the TCR-signaling (49). We then studied the usage of *SATB1* alternative promoters in Treg cells using a publicly available RNA-seq dataset [E-MTAB-2319 (16)]. As expected, Treg cells showed lower usage of P2 promoter as compared to Th2 cells. Surprisingly, P1 promoter usage was significantly higher in Treg cells (Figures 5A,B), suggesting its association with activation of TCR signaling in the absence of the pro-inflammatory cytokine signaling. To test this hypothesis further, we studied *SATB1* promoter usage in the Jurkat cell based system. We found that the *SATB1* P1 promoter usage was higher in Jurkat cells activated with P+I and that the P1 promoter usage correlated with lower SATB1 protein expression levels (Figures 5C–F). Further analysis of Ribo-Seq and RNA-Seq data from Jurkat cells revealed higher ribosome occupancy on the alternative first exons resulting from usage of the P2 and P3 promoters as compared to the P1 promoter. These results suggest differential translatability of SATB1 isoforms (Supplementary Figure 5).

## Discussion

**Figure 6:**
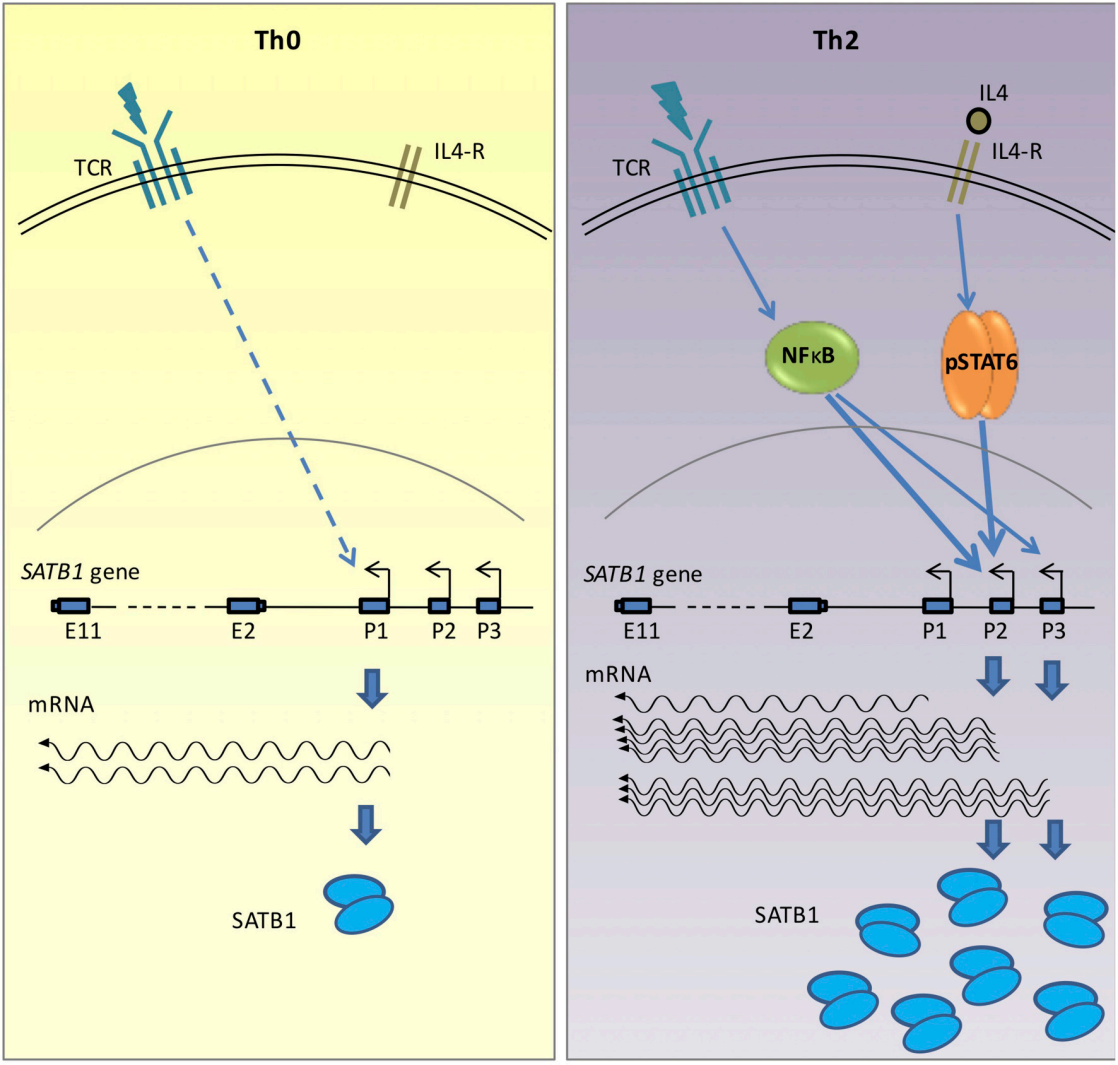
Graphical representation of *SATB1* expression via alternative promoters in activated (Th0) and polarized (Th2) CD4^+^ T-cells. Activation of non-specific TCR signaling in Th0 cells leads to *SATB1* P1 promoter usage. However, in the polarized Th2 cells, activation of cytokine signaling along with TCR activation leads to use of P2 and P3 promoters. Transcription factor STAT6, which acts downstream of the cytokine signaling and NF-κB, which acts downstream of the TCR signaling, positively regulate P2 and P3 promoter usage in polarizing conditions. The switch in promoter usage also correlates with change in SATB1 protein expression. The promoter switch may therefore enable regulation of SATB1 expression in a cell-type specific manner.

In summary, the results presented above demonstrate that *SATB1* gene expression is orchestrated by an intricate regulatory network of NF-κB signaling and cytokine signaling. During T-helper cell differentiation, the *SATB1* gene is expressed via alternative promoter usage. The P1 promoter is preferentially used by the naïve CD4^+^ T-cells and Th0 cells whereas the P2 and P3 promoters are preferentially used by the Th2 cells. STAT6 transcription factor that is downstream of cytokine signaling binds to the *SATB1* P2 promoter and positively regulates its expression in Th2 cells. The NF-κB transcription factor which is downstream of the TCR signaling also regulates the P2 and P3 promoter usage. Finally, we observed that the P1 promoter was used more in Treg cells and Th0 cells which weakly correlated with protein expression. Whether this correlation results from differential translation of resulting isoforms or differential protein degradation in different cell types needs to be further studied. The results are summarized and represented schematically in Figure 6.

Various genes have been shown to be regulated via multiple promoters during immune cell activation (50–52). Substantial number of these genes switch promoters without change in the coding DNA sequence suggesting that the promoter switch may enable their expression under different transcription factor repertoires in different physiological contexts. The master regulator transcription factor of Th2 differentiation, *GATA3* (53), and the key pro-inflammatory cytokine, *IL4* (54), have also been shown to differentially use alternative promoters in naïve and differentiated Th2 cells. Similar to *SATB1, GATA3* alternative promoters are also under the dual regulation of IL-4 and TCR signaling. However, while *GATA3* is specifically expressed in Th2 cells, the *SATB1* locus responds to cytokine signaling during both Th1 and Th2 differentiation.

T-helper cell differentiation is triggered by antigens and cytokine cues leading to activation of STAT family of transcription factors by phosphorylation. STAT4 and STAT6 play a critical role in maintaining chromatin configuration and transcription of genes that drive Th1 and Th2 differentiation, respectively. STAT4 and STAT6 bind to specific DNA sequences in these two cell types but also regulate quite a few common target genes (27), *SATB1* being one of them. STATs also shape the enhancer landscapes of T-cells. Interestingly, we did not observe expected motifs at the STAT4 and STAT6 occupancy sites in the *SATB1* P2 promoter region. The nearest direct STAT binding site was observed upstream of the *SATB1* regulatory region (data not shown). STAT6 also participates in the long-range intra-chromosomal interactions in Th2 cells to regulate expression of various gene loci (55). Therefore, the possibility that the upstream STAT binding site operates as an enhancer element to regulate P2 (and P3) promoter expression via promoter-enhancer looping cannot be ruled out.

We observed an increase in the usage of two out of the three *SATB1* alternative promoters (P2 and P3) during T-helper cell differentiation. The usage of P2 promoter increases more than that of the P3 promoter. The P3 promoter region also harbors the start site of the divergent transcript from the *SATB1-AS1* (SATB1 Anti-Sense RNA 1) gene. Bidirectional transcription is often associated with genes related to transcriptional regulation and development (56) and their promoters often coincide with large CpG islands (57). In agreement with this notion the *SATB1* P3 promoter coincides with a CpG island. However, since the usage of both P2 and P3 promoters positively correlates with SATB1 protein expression, the need for the use of two different promoters remains unclear. The use of two different promoters might presumably enable rapid increase in *SATB1* expression with an additive effect on transcription. Else, *SATB1* isoforms may affect expression and translation of other isoforms. These possibilities need to be further studied. The Jurkat cell line based system developed here can be useful for the study of promoter functions by knock-outs or similar experiments.

The Jurkat cell-line based system was also crucial in suggesting the involvement of NF-κB in *SATB1* P2 promoter usage. Jurkat cells treated with IL-4 cytokine alone did not result in phosphorylation of STAT6 or increase in P2 promoter usage (data not shown). However, IL-4 treatment in combination with activation using PMA and ionomycin (P+I) resulted in STAT6 activation and increase in P2 promoter activity. This suggested that either pSTAT6 alone is sufficient for increase in P2 promoter usage or the transcription factors downstream of the TCR signaling co-operate with STAT6 to drive the P2 promoter usage. Considering the role of NF-κB in regulation of cytokine signaling (58), we blocked NF-κB activation using chemical inhibitors and found that without affecting the global levels of STAT6 phosphorylation, the treatment resulted in reduced P2 promoter usage and also affected P3 promoter significantly in Jurkat cells. Synergistic activation of gene expression by NF-κB and STAT6 has been observed in past (43–45). Such synergism could be responsible for driving *SATB1* alternative promoter expression. Whether STAT6 and NF-κB interact directly at the P2 promoter remains to be studied.

SATB1 protein is expressed in T-helper cells in a lineage specific manner (9). Even though it is expressed in all T-helper subtypes, its role is better studied in Th2 differentiation (7). Unlike the helper T-cells, SATB1 is repressed in regulatory T-cells (11) at both transcriptional and post-transcriptional level suggesting distinct effects of pro-inflammatory cytokines and anti-inflammatory cytokines on the *SATB1* P2 promoter expression. Interestingly, even though the expression of SATB1 is suppressed in Treg cells, the P1 promoter is active. Transcription via the P1 promoter is therefore weakly correlated with SATB1 protein expression. We extended this observation to the primary CD4^+^ T-cells and Jurkat cells and observed that activation of these cells by TCR-signaling alone resulted in higher P1 promoter usage and lower SATB1 protein expression as compared to the polarizing conditions. It is therefore possible that the P1 promoter is less translatable and is used when basal level of SATB1 expression is needed.

Transcription factor/s responsible for P1 promoter usage in activated T-cells have not yet been identified. Since NF-κB inhibition had no specific effect on P1 promoter expression, AP-1 and NF-AT transcription factors both also downstream of TCR signaling, can be studied. Also, the role of NF-κB in regulation of P2 promoter usage in T-helper cells may not be direct. Further experiments need to be performed to check the direct binding of NF-κB at the P2 promoter. A co-operative binding of STAT factors with NF-κB has been previously reported (59) and cannot be ruled out in case of P2 promoter usage.

*SATB1* alternative promoter usage leads to change in the sequence of the 5' untranslated region. The 5' UTR has been shown to play a role in regulation of various genes by controlling the rate of translation via secondary structure formation, presence of upstream open reading frames, and via miRNA binding [reviewed in (60, 61)]. We observed weak correlation between P1 promoter usage and SATB1 protein expression in Treg cells and activated Jurkat cells. It would be of interest to test whether in 5′ UTR sequence differentially affects the translation efficiency of *SATB1* transcripts originating from alternative promoter usage. Further differential degradation of SATB1 protein in activated vs. polarized cells also cannot be ruled out as a cause of this discrepancy.

In summary, we have elucidated a complex mode of regulation of *SATB1* gene expression via alternative promoters during peripheral differentiation of T-cells. *SATB1* regulation represents a unique example with conserved mode of regulation in multiple T-helper subtypes with possible effect on protein levels. We anticipate that understanding of the unique functions of these promoters in gene regulation and the physiological consequences of their expression/repression will be a prime focus of the future studies.

## Data Availability

The datasets generated for this study can be found in NCBI SRA, PRJNA503398.

## Ethics Statement

This study was carried out in accordance with the recommendations of the Institutional Human Ethics Committee, IISER-Pune with written informed consent from all subjects. All subjects gave written informed consent. The protocol was approved by the Institutional Human Ethics Committee, IISER-Pune.

Mice used in this study were maintained in the Central Animal Laboratory at Turku University. This study was carried out in accordance with appropriate guidelines for the care and use of laboratory animals and were approved by the “Finnish Animal Ethics Committee.” The protocol was approved by the “Finnish Animal Ethics Committee.”

## Author Contributions

SK designed experiments, performed ChIP-Seq and RNA-Seq analysis, performed Jurkat cell-line experiments, T-helper cell differentiation, qRT-PCR analysis, and wrote the manuscript. AS and IP performed FACS staining, *Stat4/Stat6* KO experiments, performed T-helper cell differentiation, and qRT-PCR analysis. RB performed Jurkat cell-line experiments. AVS assisted in Jurkat cell experiments and T-helper cell differentiation. PR assisted in RNA-Seq analysis. ZC provided expertise in *Stat4/Stat6* KO mouse experiments, RL participated in experimental design, discussed and interpreted results and supported the research. SG designed the experiments, was involved in regular discussions and data interpretations, supported the research and wrote the manuscript.

### Conflict of Interest Statement

The authors declare that the research was conducted in the absence of any commercial or financial relationships that could be construed as a potential conflict of interest.

## References

[1] Hosokawa H Rothenberg EV. Cytokines, transcription factors, and the initiation of T-cell development. Cold Spring Harb Perspect Biol. (2018) 10:a028621. 10.1101/cshperspect.a028621 28716889 PMC5876153

[2] Hoefig KP Heissmeyer V. Posttranscriptional regulation of T helper cell fate decisions. J Cell Biol. (2018) 217:2615–31. 10.1083/jcb.201708075 29685903 PMC6080923

[3] Zhu J Paul WE. Peripheral CD4+ T-cell differentiation regulated by networks of cytokines and transcription factors. Immunol Rev. (2010) 238:247–62. 10.1111/j.1600-065X.2010.00951.x 20969597 PMC2975272

[4] Dickinson LA Joh T Kohwi Y Kohwi-Shigematsu T. A tissue-specific MAR/SAR DNA-binding protein with unusual binding site recognition. Cell. (1992) 70:631–45. 10.1016/0092-8674(92)90432-C 1505028

[5] Burute M Gottimukkala K Galande S. Chromatin organizer SATB1 is an important determinant of T-cell differentiation. Immunol Cell Biol. (2012) 90:852–9. 10.1038/icb.2012.28 22710879

[6] Cai S Lee CC Kohwi-Shigematsu T. SATB1 packages densely looped, transcriptionally active chromatin for coordinated expression of cytokine genes. Nat Genet. (2006) 38:1278–88. 10.1038/ng1913 17057718

[7] Ahlfors H Limaye A Elo LL Tuomela S Burute M Gottimukkala KV. SATB1 dictates expression of multiple genes including IL-5 involved in human T helper cell differentiation. Blood. (2010) 116:1443–53. 10.1182/blood-2009-11-252205 20522714 PMC2938835

[8] Alvarez JD Yasui DH Niida H Joh T Loh DY Kohwi-Shigematsu T. The MAR-binding protein SATB1 orchestrates temporal and spatial expression of multiple genes during T-cell development. Genes Dev. (2000) 14:521–35. 10.1101/gad.14.5.521 10716941 PMC316425

[9] Kakugawa K Kojo S Tanaka H Seo W Endo TA Kitagawa Y. Essential roles of SATB1 in specifying T lymphocyte subsets. Cell Rep. (2017) 19:1176–88. 10.1016/j.celrep.2017.04.038 28494867

[10] Notani D Gottimukkala KP Jayani RS Limaye AS Damle MV Mehta S. Global regulator SATB1 recruits beta-catenin and regulates T(H)2 differentiation in Wnt-dependent manner. PLoS Biol. (2010) 8:e1000296. 10.1371/journal.pbio.1000296 20126258 PMC2811152

[11] Beyer M Thabet Y Müller RU Sadlon T Classen S Lahl K. Repression of the genome organizer SATB1 in regulatory T cells is required for suppressive function and inhibition of effector differentiation. Nat Immunol. (2011) 12:898–907. 10.1038/ni.2084 21841785 PMC3669688

[12] Kondo M Tanaka Y Kuwabara T Naito T Kohwi-Shigematsu T Watanabe A SATB1 plays a critical role in establishment of immune tolerance. J Immunol. (2016) 196:563–72. 10.4049/jimmunol.1501429 26667169

[13] Kitagawa Y Ohkura N Kidani Y Vandenbon A Hirota K Kawakami R Guidance of regulatory T cell development by Satb1-dependent super-enhancer establishment. Nat Immunol. (2017) 18:173–83. 10.1038/ni.3646 27992401 PMC5582804

[14] Gottimukkala KP Jangid R Patta I Sultana DA Sharma A Misra-Sen J. Regulation of SATB1 during thymocyte development by TCR signaling. Mol Immunol. (2016) 77:34–43. 10.1016/j.molimm.2016.07.005 27454343 PMC6612261

[15] Davuluri RV Suzuki Y Sugano S Plass C Huang TH. The functional consequences of alternative promoter use in mammalian genomes. Trends Genet. (2008) 24:167–77. 10.1016/j.tig.2008.01.008 18329129

[16] Ranzani V Rossetti G Panzeri I Arrigoni A Bonnal RJ Curti S. The long intergenic noncoding RNA landscape of human lymphocytes highlights the regulation of T cell differentiation by linc-MAF-4. Nat Immunol. (2015) 16:318–25. 10.1038/ni.3093 25621826 PMC4333215

[17] Martinez NM Pan Q Cole BS Yarosh CA Babcock GA Heyd F. Alternative splicing networks regulated by signaling in human T cells. RNA. (2012) 18:1029–40. 10.1261/rna.032243.112 22454538 PMC3334690

[18] Kanduri K Tripathi S Larjo A Mannerström H Ullah U Lund R. Identification of global regulators of T-helper cell lineage specification. Genome Med. (2015) 7:122. 10.1186/s13073-015-0237-0 26589177 PMC4654807

[19] Harrow J Frankish A Gonzalez JM Tapanari E Diekhans M Kokocinski F. GENCODE: the reference human genome annotation for the ENCODE project. Genome Res. (2012) 22:1760–74. 10.1101/gr.135350.111 22955987 PMC3431492

[20] Pertea M Kim D Pertea GM Leek JT Salzberg SL. Transcript-level expression analysis of RNA-seq experiments with HISAT, StringTie and Ballgown. Nat Protoc. (2016) 11:1650–67. 10.1038/nprot.2016.095 27560171 PMC5032908

[21] Thorvaldsdóttir H Robinson JT Mesirov JP. Integrative Genomics Viewer (IGV): high-performance genomics data visualization and exploration. Brief Bioinform. (2013) 14:178–92. 10.1093/bib/bbs017 22517427 PMC3603213

[22] Karolchik D Hinrichs AS Furey TS Roskin KM Sugnet CW Haussler D. The UCSC table browser data retrieval tool. Nucleic Acids Res. (2004) 32:D493–6. 10.1093/nar/gkh103 14681465 PMC308837

[23] Liao Y Smyth GK Shi W. featureCounts: an efficient general purpose program for assigning sequence reads to genomic features. Bioinformatics. (2013) 30:923–30. 10.1093/bioinformatics/btt656 24227677

[24] Love MI Huber W Anders S. Moderated estimation of fold change and dispersion for RNA-seq data with DESeq2. Genome Biol. (2014) 15:550. 10.1186/s13059-014-0550-8 25516281 PMC4302049

[25] Robinson MD McCarthy DJ Smyth GK. edgeR: a bioconductor package for differential expression analysis of digital gene expression data. Bioinformatics. (2010) 26:139–40. 10.1093/bioinformatics/btp616 19910308 PMC2796818

[26] Statistical Significance of Overlap of Two Groups of Genes Available online at: http://nemates.org/MA/progs/overlap_stats.html (accessed March 19, 2019).

[27] Wei L Vahedi G Sun HW Watford WT Takatori H Ramos HL. Discrete roles of STAT4 and STAT6 transcription factors in tuning epigenetic modifications and transcription during T helper cell differentiation. Immunity. (2010) 32:840–51. 10.1016/j.immuni.2010.06.003 20620946 PMC2904651

[28] Wei G Wei L Zhu J Zang C Hu-Li J Yao Z. Global mapping of H3K4me3 and H3K27me3 reveals specificity and plasticity in lineage fate determination of differentiating CD4+ T cells. Immunity. (2009) 30:155–67. 10.1016/j.immuni.2008.12.009 19144320 PMC2722509

[29] Langmead B Salzberg SL. Fast gapped-read alignment with Bowtie 2. Nat Methods. (2012) 9:357–9. 10.1038/nmeth.1923 22388286 PMC3322381

[30] Li H Durbin R. Fast and accurate long-read alignment with Burrows-Wheeler transform. Bioinformatics. (2010) 26:589–95. 10.1093/bioinformatics/btp698 20080505 PMC2828108

[31] Zhang Y Liu T Meyer CA Eeckhoute J Johnson DS Bernstein BE. Model-based analysis of ChIP-Seq (MACS). Genome Biol. (2008) 9:R137. 10.1186/gb-2008-9-9-r137 18798982 PMC2592715

[32] Schneider CA Rasband WS Eliceiri KW. NIH Image to ImageJ: 25 years of image analysis. Nat Methods. (2012) 9:671–5. 10.1038/nmeth.2089 22930834 PMC5554542

[33] Santos-Rosa H Schneider R Bannister AJ Sherriff J Bernstein BE Emre NC. Active genes are tri-methylated at K4 of histone H3. Nature. (2002) 419:407–11. 10.1038/nature01080 12353038

[34] Zheng W Flavell RA. The transcription factor GATA-3 is necessary and sufficient for Th2 cytokine gene expression in CD4 T cells. Cell. (1997) 89:587–96. 10.1016/S0092-8674(00)80240-8 9160750

[35] Wei G Abraham BJ Yagi R Jothi R Cui K Sharma S. Genome-wide analyses of transcription factor GATA3-mediated gene regulation in distinct T cell types. Immunity. (2011) 35:299–311. 10.1016/j.immuni.2011.08.007 21867929 PMC3169184

[36] Kaplan MH Schindler U Smiley ST Grusby MJ. Stat6 is required for mediating responses to IL-4 and for development of Th2 cells. Immunity. (1996) 4:313–9. 10.1016/S1074-7613(00)80439-2 8624821

[37] Seif F Khoshmirsafa M Aazami H Mohsenzadegan M Sedighi G Bahar M. The role of JAK-STAT signaling pathway and its regulators in the fate of T helper cells. Cell Commun Signal. (2017) 15:23. 10.1186/s12964-017-0177-y 28637459 PMC5480189

[38] Jacobson NG Szabo SJ Weber-Nordt RM Zhong Z Schreiber RD Darnell JE. Interleukin 12 signaling in T helper type 1 (Th1) cells involves tyrosine phosphorylation of signal transducer and activator of transcription (Stat)3 and Stat4. J Exp Med. (1995) 181:1755–62. 10.1084/jem.181.5.1755 7722452 PMC2191986

[39] Chatila T Silverman L Miller R Geha R. Mechanisms of T cell activation by the calcium ionophore ionomycin. J Immunol. (1989) 143:1283–9. 2545785

[40] Timmerman LA Clipstone NA Ho SN Northrop JP Crabtree GR. Rapid shuttling of NF-AT in discrimination of Ca2+ signals and immunosuppression. Nature. (1996) 383:837–40. 10.1038/383837a0 8893011

[41] Rincón M Flavell RA. AP-1 transcriptional activity requires both T-cell receptor-mediated and co-stimulatory signals in primary T lymphocytes. EMBO J. (1994) 13:4370–81. 10.1002/j.1460-2075.1994.tb06757.x 7925281 PMC395364

[42] Verweij CL Geerts M Aarden LA. Activation of interleukin-2 gene transcription via the T-cell surface molecule CD28 is mediated through an NF-kB-like response element. J Biol Chem. (1991) 266:14179–82. 1650350

[43] Shen CH Stavnezer J. Interaction of stat6 and NF-kappaB: direct association and synergistic activation of interleukin-4-induced transcription. Mol Cell Biol. (1998) 18:3395–404. 10.1128/MCB.18.6.3395 9584180 PMC108921

[44] Goto K Chiba Y Matsusue K Hattori Y Maitani Y Sakai H. The proximal STAT6 and NF-kappaB sites are responsible for IL-13- and TNF-alpha-induced RhoA transcriptions in human bronchial smooth muscle cells. Pharmacol Res. (2010) 61:466–72. 10.1016/j.phrs.2009.12.001 20006706 PMC3486725

[45] Schaffer A Cerutti A Shah S Zan H Casali P. The evolutionarily conserved sequence upstream of the human Ig heavy chain S gamma 3 region is an inducible promoter: synergistic activation by CD40 ligand and IL-4 via cooperative NF-kappa B and STAT-6 binding sites. J Immunol. (1999) 162:5327–36. 10228008

[46] Ballard DW Böhnlein E Lowenthal JW Wano Y Franza BR Greene WC. HTLV-I tax induces cellular proteins that activate the kappa B element in the IL-2 receptor alpha gene. Science. (1988) 241:1652–5. 10.1126/science.2843985 2843985

[47] Das J Chen CH Yang L Cohn L Ray P Ray A. A critical role for NF-kappa B in GATA3 expression and TH2 differentiation in allergic airway inflammation. Nat Immunol. (2001) 2:45–50. 10.1038/83158 11135577

[48] Chen W Jin W Hardegen N Lei KJ Li L Marinos N. Conversion of peripheral CD4+CD25- naive T cells to CD4+CD25+ regulatory T cells by TGF-beta induction of transcription factor Foxp3. J Exp Med. (2003) 198:1875–86. 10.1084/jem.20030152 14676299 PMC2194145

[49] Ruan Q Kameswaran V Tone Y Li L Liou HC Greene MI. Development of Foxp3(+) regulatory t cells is driven by the c-Rel enhanceosome. Immunity. (2009) 31:932–40. 10.1016/j.immuni.2009.10.006 20064450 PMC2807990

[50] Morgan MA Magnusdottir E Kuo TC Tunyaplin C Harper J Arnold SJ. Blimp-1/Prdm1 alternative promoter usage during mouse development and plasma cell differentiation. Mol Cell Biol. (2009) 29:5813–27. 10.1128/MCB.00670-09 19737919 PMC2772737

[51] Gómez-del Arco P Kashiwagi M Jackson AF Naito T Zhang J Liu F Alternative promoter usage at the Notch1 locus supports ligand-independent signaling in T cell development and leukemogenesis. Immunity. (2011) 33:685–98. 10.1016/j.immuni.2010.11.008 PMC3072037 21093322

[52] Takadera T Leung S Gernone A Koga Y Takihara Y Miyamoto NG. Structure of the two promoters of the human lck gene: differential accumulation of two classes of lck transcripts in T cells. Mol Cell Biol. (1989) 9:2173–80. 10.1128/MCB.9.5.2173 2787474 PMC363011

[53] Scheinman EJ Avni O. Transcriptional regulation of GATA3 in T helper cells by the integrated activities of transcription factors downstream of the interleukin-4 receptor and T cell receptor. J Biol Chem. (2009) 284:3037–48. 10.1074/jbc.M807302200 19056736

[54] Li B Tournier C Davis RJ Flavell RA. Regulation of IL-4 expression by the transcription factor JunB during T helper cell differentiation. EMBO J. (1999) 18:420–32. 10.1093/emboj/18.2.420 9889198 PMC1171136

[55] Spilianakis CG Flavell RA. Long-range intrachromosomal interactions in the T helper type 2 cytokine locus. Nat Immunol. (2004) 5:1017–27. 10.1038/ni1115 15378057

[56] Lepoivre C Belhocine M Bergon A Griffon A Yammine M Vanhille L. Divergent transcription is associated with promoters of transcriptional regulators. BMC Genomics. (2013) 14:914. 10.1186/1471-2164-14-914 24365181 PMC3882496

[57] Trinklein ND Aldred SF Hartman SJ Schroeder DI Otillar RP Myers RM. An abundance of bidirectional promoters in the human genome. Genome Res. (2004) 14:62–6. 10.1101/gr.1982804 14707170 PMC314279

[58] Khalaf H Jass J Olsson PE. Differential cytokine regulation by NF-kappaB and AP-1 in Jurkat T-cells. BMC Immunol. (2010) 11:26. 10.1186/1471-2172-11-26 20507572 PMC2889865

[59] Ganster RW Taylor BS Shao L Geller DA. Complex regulation of human inducible nitric oxide synthase gene transcription by Stat 1 and NF-kappa B. Proc Natl Acad Sci USA. (2001) 98:8638–43. 10.1073/pnas.151239498 11438703 PMC37488

[60] Hinnebusch AG Ivanov IP Sonenberg N. Translational control by 5'-untranslated regions of eukaryotic mRNAs. Science. (2016) 352:1413–6. 10.1126/science.aad9868 27313038 PMC7422601

[61] O'Brien J Hayder H Zayed Y Peng C. Overview of microRNA biogenesis, mechanisms of actions, and circulation. Front Endocrinol. (2018) 9:402. 10.3389/fendo.2018.00402 30123182 PMC6085463

