# Supplementary material for: NF-κB Signaling and IL-4 Signaling Regulate SATB1 Expression via Alternative Promoter Usage During Th2 Differentiation
Source: Front Immunol. 2019 Apr 2;10:667. doi: 10.3389/fimmu.2019.00667 (PMC6454056; doi:10.3389/fimmu.2019.00667)
Supplement: Supplementary file 1 [file Data_Sheet_1.docx]

**Supplementary data**

**NF-κB signaling and IL-4 signaling regulate SATB1 expression via alternative promoter usage during Th2 differentiation**

**Authors**: Satyajeet Khare, Ankitha Shetty, Rahul Biradar, Indumathi Patta, Zhi Jane Chen, Ameya Sathe, Puli Chandramouli Reddy, Riitta Lahesmaa, Sanjeev Galande*

* Correspondence:

Sanjeev Galande

[sanjeev@iiserpune.ac.in](mailto:sanjeev@iiserpune.ac.in)

**Supplementary methods**:

**Flow Cytometry**:

For CD4 staining, PBMCs and naive CD4 T cells were washed once with PBS and stained with anti-Human CD4 PE antibody (555347, BD Biosciences) as per manufacturer’s instructions. After staining, cells were fixed with 1% formaldehyde at room temperature for 15 minutes, washed again with PBS and resuspended in FACS buffer (PBS with 5% fetal bovine serum) before analysis on flow cytometer. For intracellular cytokine staining, naive CD4 cells were *in vitro* differentiated into Th2 cells. Cells were then harvested and were permeabilized on ice for 15 minutes followed by staining with anti-Human IL4 antibody (12-7049-42, eBioscience) as per manufacturer’s instructions in the presence of BD GolgiPlug (555029, BD Biosciences). Cells were then resuspended in FACS buffer until analysis.

**RNA-Seq Analysis**:

Publicly available mouse CD4+ T-cell polyA RNA-Seq dataset (GSE48138 [1]) was analyzed to identify *Satb1* expression in various T helper cell subtypes. In brief, reads were aligned to reference mouse genome assembly (mm10, Gencode) using HiSAT2 [2]. *Satb1* expression was analyzed in naive, Th1, Th2, Th17 and induced Treg (iTreg) cells using DESeq2 [3].

***Stat4* and *Stat6* knockout animals**:

Naïve CD4+ cells isolated from spleens of WT and *Stat4* or *Stat6* KO Balb/c mice (CD4+CD62L+ T cell Isolation Kit II, Miltenyi Biotec, 130-093-227) were subjected to CD4 and CD62L staining to confirm purity (Fig 2A). A fraction of naïve CD4+ T-cells was activated in presence of plate bound anti-CD28 (BD 553295), plate bound anti-CD3 (BD 553238), IL-2 (R&D 419 402-ML) or subjected to in vitro Th1 differentiation in additional presence of IL-12 (R&D 419-ML) and anti-IL4 (BD 554432). Th2 differentiation was performed as described in the manuscript. Th1 and Th2 differentiation was confirmed by IFN-γ (BD 554411) and GATA3 (BD 560074) staining respectively. Remaining cells were used for RNA isolation followed by qRT-PCR analysis.

**Satb1 expression in *Stat4* KO mice**:

Spleen and lymph nodes from wild type and *Stat4* knock-out mice were dissected. The cells were extracted and subjected to naïve cell isolation. A fraction of naïve cells was subjected to Th1 differentiating conditions. Naïve and differentiated cells were either used for RNA isolation followed by qRT-PCR analysis or were subjected to flow cytometry analysis for Satb1 protein expression (please refer to the methods section of the main manuscript for details).

**Translatability of alternative transcripts**:

RNA-Seq (GSM2171783[4]) and Ribo-Seq (GSM1916182[5]) data were analyzed to compare translatability of the SATB1 isoforms. In brief, Bedgraph and BigWigfiles were downloaded from GEO database for Ribo-Seq and RNA-Seq data respectively. Ribo-Seq data was converted from Bedgraph format to Bigwig format using bedGraphToBigWig [6]. The BigWig files were uploaded on IGV [7] using hg19 genome assembly. MultiBigWigSummary [8] was used to generate average scores for genomic regions of interest (alternative first exons ENSE00001376874, ENSE00001728511 and ENSE00001884403 corresponding to *SATB1* P1, P2 and P3 promoters respectively). Ratio of counts generated by Ribo-Seq data and RNA-Seq data were plotted as a measure of ribosome binding (translatability) of the exons and thus alternative transcripts.

**Supplementary Results**:

**Isolation and differentiation of CD4 T-cells**:

We performed flow cytometry of surface marker CD4 to confirm naive CD4 T-cell isolation from PBMC (Supp. Fig 1A-1B). The naive CD4 T-cells that were subjected to Th2 differentiating conditions showed higher intracellular IL-4 levels confirming Th2 differentiation (Supp. Fig 1C-1D).


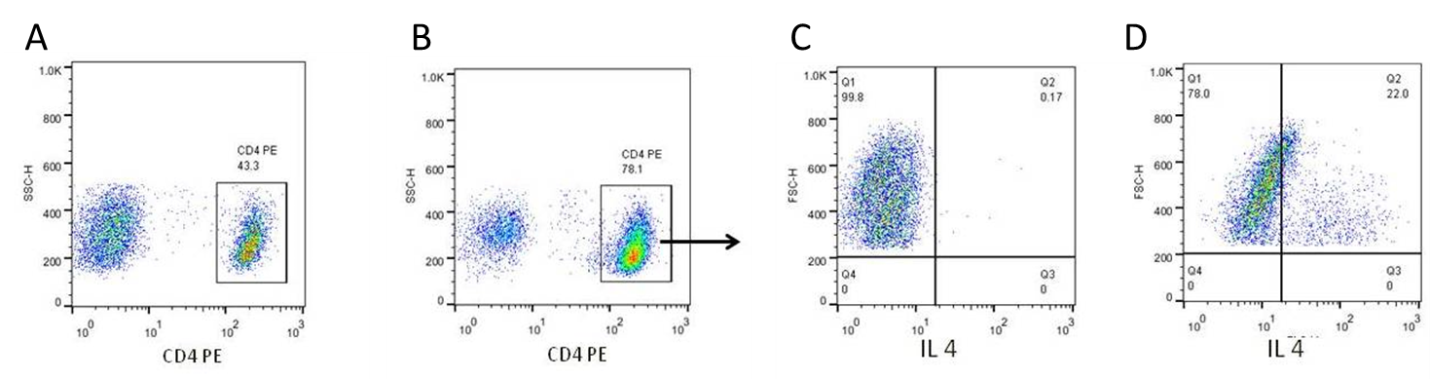


**Supplementary figure 1: Flow cytometry analysis of human naive CD4 T-cell isolation and Th2 differentiation**. **(A-B)** Flow cytometry of peripheral blood mononuclear cells (PBMCs) isolated from healthy human volunteers. Naive CD4 + T cells isolated (A) and stimulated with anti-CD3 and anti-CD28 for 96 hr to differentiate into Th2 cells (B). Th2 differentiation is confirmed by IL4 staining of naive CD4+ T cells and Th2 cells. **(C-D)** Increase in IL4 producing cell population observed upon differentiation conditions.

***Stat4*/*Stat6* knockout mice**:

We performed flow cytometry for CD4-CD62L expression to confirm the purity of naïve cells isolated from murine spleens (Supp. Fig 2A). Naïve CD4 cells were further subjected to Th1 and Th2 differentiation *in vitro*. The differentiation was confirmed based on IFN-γ and Gata3 staining respectively (Fig 2B-2E). The T-helper cell differentiation was severely affected in knockout mice. *Stat4* and *Stat6* knockout mice showed very small percentage of IFN-γ and Gata3 expressing cells respectively as compared to the wild-type mice.


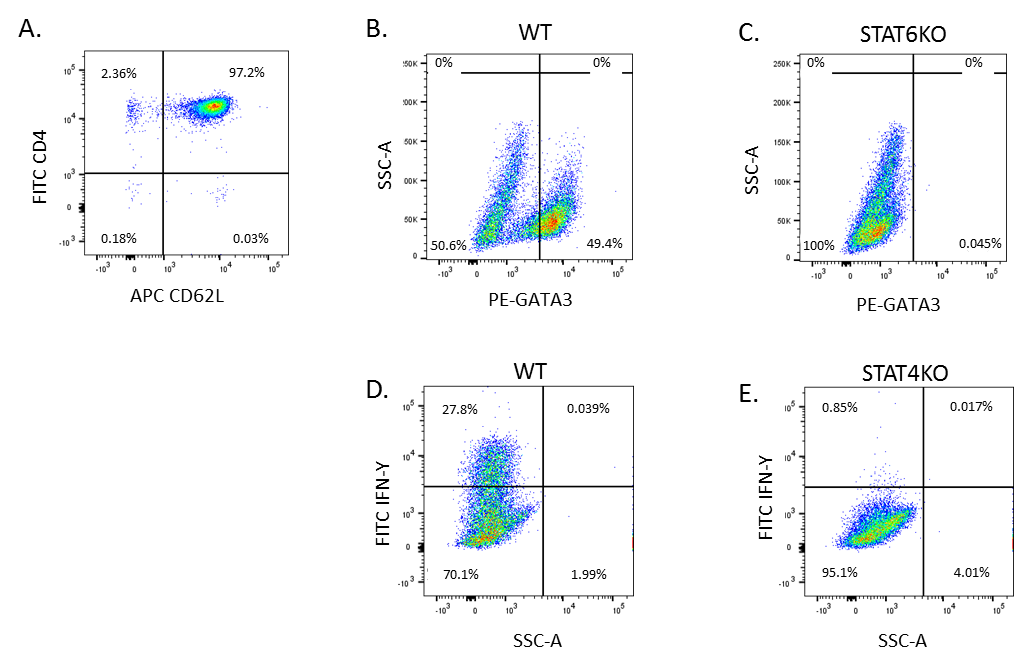


**Supplementary figure 2: Flow cytometry analysis of murine naïve CD4 T-cell isolation and Th1/Th2 differentiation. (A)** CD4-CD62L flow cytometry analysis confirming purity of naïve CD4+ cell isolation from WT Balb/c mice **(B – C)** Gata3 staining to confirm Th2 differentiation (day 5) in CD4+ cells isolated from WT and STAT6 KO Balb/c mice. **(D – E)** IFN-γ staining to confirm Th1 differentiation (day 5) in CD4+ cells isolated from WT and STAT4 KO Balb/c mice.

**Satb1 expression in various CD4 T-cells**:

We analyzed publically available transcriptome data (GSE48138) as described above for the expression of *Satb1*gene in different CD4+ T-cells (Supp. Fig 3). Higher levels of *Satb1* gene expression were observed in T-helper cells as compared to regulatory T-cells suggesting opposing effects of pro-inflammatory cytokines and anti-inflammatory cytokines on *Satb1* expression. We then studied *Satb1* promoter expression in T-helper cells and Treg cells and found that high P2 and P3 expression was a characteristic of T-helper cells. Interestingly, higher P1 promoter expression was observed in Treg cells (Fig 5B, main text).


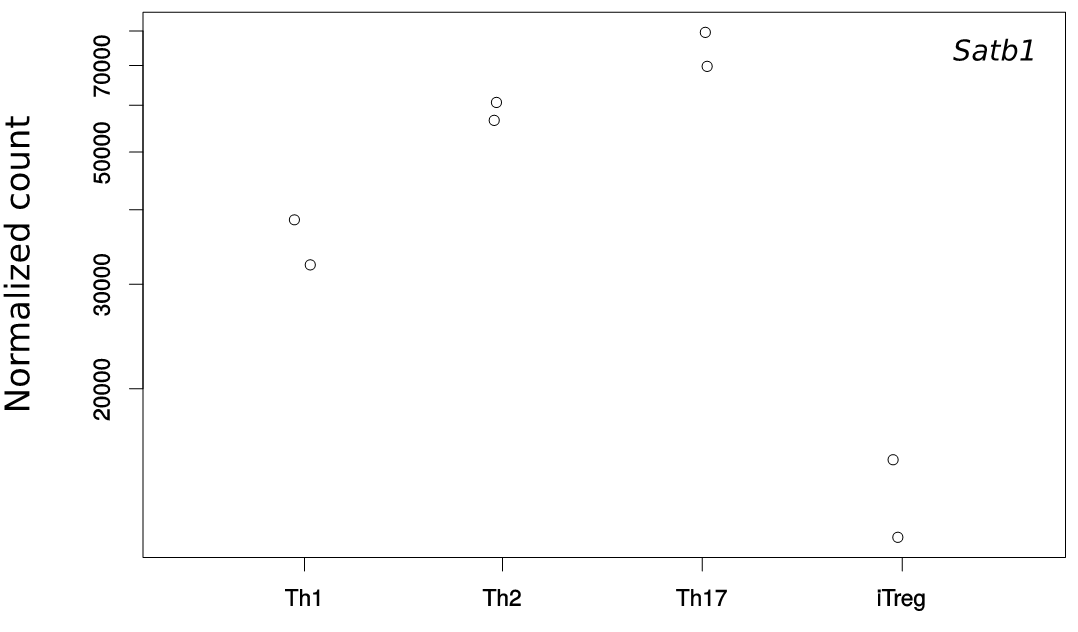


**Supplementary figure 3: *Satb1* alternative promoters in various CD4+ T-cells**. Available transcriptome data­ (GSE48138) was analyzed for total *Satb1* expression in Th1, Th2, Th17 and iTreg cells. *Satb1* expression was plotted as normalized counts in various CD4+ T-cell subtypes. *Satb1* expression was high in T-helper cells as compared to regulatory T-cells suggesting a possible role of pro-inflammatory cytokines in *Satb1* expression.

***Satb1* alternative promoter usage in Stat4 KO animals**:

We analyzed ChIP-Seq data for master regulator transcription factors of Th1 differentiation, Stat4 (GSE22105) for their involvement in *Satb1* promoter regulation. We found that Stat4 showed differential occupancy on mouse *Satb1* promoters. Interestingly, Stat4 occupied P2 regions but not the P1 and P3 promoter (Supp. Fig. 4A). To study the importance of Stat4 binding in the regulation of alternative promoter usage, we used *Stat4*-KO mice. Naïve CD4+ T-cells isolated from spleen and lymph nodes from wild-type and *Stat4*-KO mice were subjected to Th1 differentiation conditions. *Satb1* isoform expression analysis by qRT-PCR suggested that P2 and P3 promoter usage was significantly affected in *Stat4*-KO mice (Supp. Fig. 4B). *Stat4*-KO also resulted in a significant decrease in Satb1 protein expression (Supp. Fig. 4C) suggesting a significant contribution of P2 and P3 promoters towards protein production.


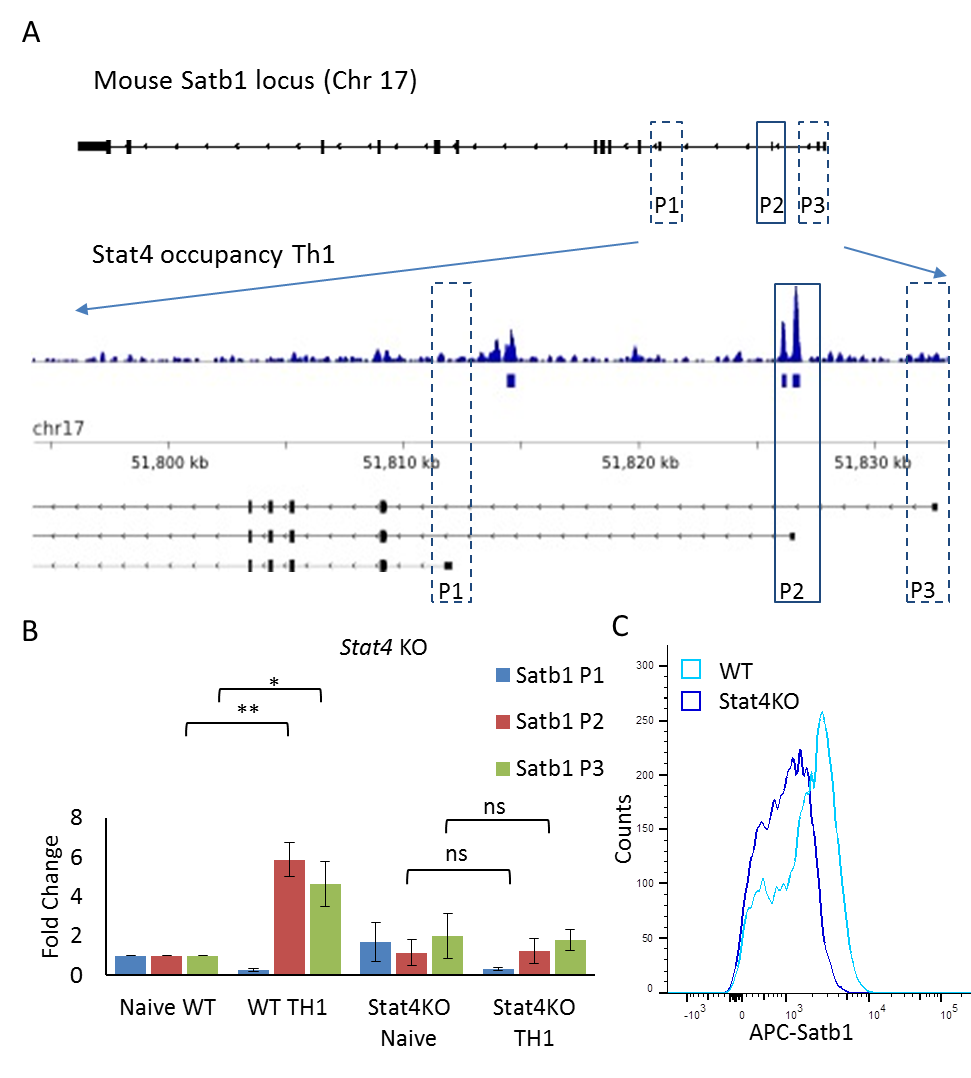


**Supplementary figure 4: Stat4 regulates Satb1 P2 promoter expression in vivo. (A)** ChIP-Seq analysis of Stat4 occupancy at the *Satb1* alternative promoters in Th1 cells (*Satb1* regulatory region is zoomed in). Stat4 ChIP-Seq aligned reads (first track) and significant peaks (second track) along with *Satb1* alternative promoters in mouse (mm10 genome assembly). Stat4 binds to the *Satb1* P2 promoter in T-helper cells. **(B)** qRT-PCR analysis of *Satb1* alternative promoter usage (P1, P2 and P3) in naive CD4+ and Th1 cells performed in WT and *Stat4* KO mice respectively. Error bar represents standard deviation (N=3); P-values calculated using student t test (*>0.1, **>0.05). *Stat4* KO adversely affects the *Satb1* alternative promoter usage. Unlike the wild type animals, no significant increase is observed in *Satb1* P2 and P3 promoter usage in cells from *Stat4* KO animals subjected to Th1 differentiation conditions. **(C)** Flow cytometry analysis for Satb1 protein expression in wild-type and *Stat4* KO animals respectively. Satb1 protein expression also does not increase when naive T-cells from *Stat4* KO animals were subjected to Th1 differentiation conditions.

**Differential translatability of *SATB1* isoforms**:

We observed that *SATB1* alternative promoter usage during T-cell activation correlated with differential protein expression. Specifically, P1 promoter which is predominantly used by activated T-cells showed weak correlation with SATB1 protein expression (Fig 5F). To study if *SATB1* alternative isoforms show differential translatability, we analyzed publicly available Ribo-Seq (determinant of active translation) data against RNA-Seq data from resting Jurkat cells. We observed that, though alternative first exon corresponding to the P1 promoter harbors more RNA-Seq reads, alternative first exons corresponding to P2 and P3 promoters show more ribosome occupancy and thereby, more translatability. (Supp. Fig. 5A and 5B). These results, however, need to be validated using additional biological replicates. Additionally, the role of differential protein degradation leading to differential protein amounts in resting and activated T-cells needs to be tested.

**
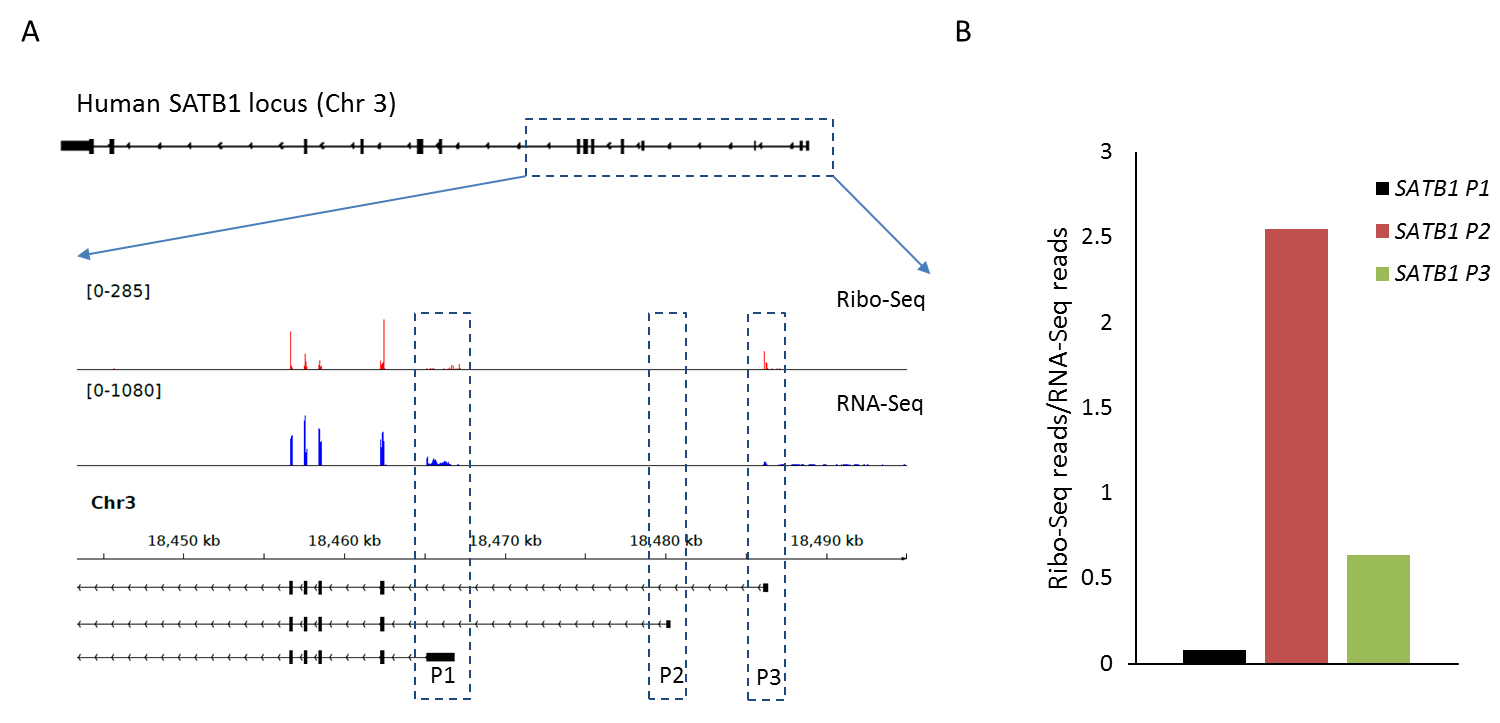
**

**Supplementary figure 5: Differential translatability of the *SATB1* P1, P2 and P3 transcripts. (A)** Ribo-Seq (GSM1916182) and RNA-Seq (GSM2171783) analysis of Jurkat cells (*SATB1* regulatory region is zoomed in). Ribo-Seq reads predominate at P2 and P3 promoters (first track) whereas RNA-Seq reads predominate at the P1 promoter (second track) of the *SATB1* gene (hg19 genome assembly). **(B)** A ratio of the aligned reads obtained at the alternative first exons of *SATB1* gene. Alternative exons that correspond to P2 and P3 promoters shows higher ratio of Ribo-Seq reads over RNA-Seq reads suggesting higher translatability.

**Supplementary Table1: List of primers used for qRT-PCR analysis**.

| **Primer** | **Sequence (5’ to 3’)** |
| --- | --- |
| Hs_SATB1_E1a_Fw (P1) | CCTTCAGGTCTGCTGCTTTT |
| Hs_SATB1_E1b_Fw (P2) | TGCTAGCAGTGCCAGAGAGA |
| Hs_SATB1_E1c_Fw (P3) | AGCCGTTCTTGGTTTCAGG |
| Hs_SATB1_E2_Rv | CCCTTCGGATCACTCACATT |
| Hs_SATB1_E2_Fw (Ctrl) | GAAGAGGAAGGCTTGGGAGT |
| Hs_SATB1_E3_Rv (Ctrl) | ATGCTCCTCCTTGCAATCAT |
| Hs_18s_rRNA_Fw | CGCCGCTAGAGGTGAAATTCT |
| Hs_18s_rRNA_Rv | CGAACCTCCGACTTTCGTTCT |
| Hs_IL2RA_Fw | TACCTGCTGATGTGGGGACT |
| Hs_IL2RA_Rv | TAGGCCATGGCTTTGAATGT |
| Hs_GATA3_Fw | TCATTAAGCCCAAGCGAAGG |
| Hs_GATA3_Rv | GTCCCCATTGGCATTCCTC |
| Mm_SATB1_E1a_Fw_P1 | CAAGAATCCCGGCTGCAAAG |
| Mm_SATB1_E2_Rv_P1 | CCCTGAGTTGCCTCGTTCAA |
| Mm_SATB1_E1b_Fw_P2 | AGATTCGGAAACCAGCCTCTG |
| Mm_SATB1_E2_Rv_P2 | GGACCCTTCGGATCACTCAC |
| Mm_SATB1_E1c_Fw_P3 | CGGTTCCACGCCTGATTC |
| Mm_SATB1_E2_Rv_P3 | GTGGACCCTTCGGATCACTC |
| Mm_SATB1_E3_Fw (Ctrl) | TGATAGAGATGGCGTTGCTG |
| Mm_SATB1_E4_Rv (Ctrl) | TTTTGAGGGTGACCACATGA |
| Mm_18s_rRNA_Fw | GTAACCCGTTGAACCCCATT |
| Mm_18s_rRNA_Rv | CCATCCAATCGGTAGTAGCG |

**Supplementary references**:

1. Hu, G., et al., *Expression and regulation of intergenic long noncoding RNAs during T cell development and differentiation.* Nat Immunol, 2013. **14**(11): p. 1190-8.

2. Pertea, M., et al., *Transcript-level expression analysis of RNA-seq experiments with HISAT, StringTie and Ballgown.* Nat Protoc, 2016. **11**(9): p. 1650-67.

3. Love, M.I., W. Huber, and S. Anders, *Moderated estimation of fold change and dispersion for RNA-seq data with DESeq2.* Genome Biol, 2014. **15**(12): p. 550.

4. Liau, W.S., et al., *Aberrant activation of the GIMAP enhancer by oncogenic transcription factors in T-cell acute lymphoblastic leukemia.* Leukemia, 2017. **31**(8): p. 1798-1807.

5. Gawron, D., et al., *Positional proteomics reveals differences in N-terminal proteoform stability.* Mol Syst Biol, 2016. **12**(2): p. 858.

6. Kent, W.J., et al., *BigWig and BigBed: enabling browsing of large distributed datasets.* Bioinformatics, 2010. **26**(17): p. 2204-7.

7. Thorvaldsdottir, H., J.T. Robinson, and J.P. Mesirov, *Integrative Genomics Viewer (IGV): high-performance genomics data visualization and exploration.* Brief Bioinform, 2013. **14**(2): p. 178-92.

8. Ramirez, F., et al., *deepTools2: a next generation web server for deep-sequencing data analysis.* Nucleic Acids Res, 2016. **44**(W1): p. W160-5.
